# Improvisation, the butterfly and the cultivation of wonder

**DOI:** 10.1111/medu.15675

**Published:** 2025-04-15

**Authors:** Christiaan Alexander Rhodius, Marco Antonio de Carvalho Filho

**Affiliations:** ^1^ Hospice Bardo – Hospice Hoofddorp Netherlands; ^2^ University Medical Centre Groningen Thoraxcentre ‐ Center for Education Development and Research in Health Professions Groningen Netherlands

## Abstract

In a complex world, healthcare needs improvisation. The authors reflect on the importance of safe, emotion‐aware, learning along with how relational safety and wonder makes improvisation vital, routine, and collaborative.

The increasing complexity of an ever‐changing world impacts the way healthcare is delivered, be it through pandemics, effects of climate change or digital accessibility to (dis)information^1^ Medical education should embrace this complexity^2^, ^3^ because we need a health workforce that is able to improvise if it is to provide socially just healthcare. Improvisation stems from ‘improvisus’, that which is unforeseen (or unseen).^4^ Using improvisation in education could help nurture the necessary attitude for dealing with complexity by actively creating experience working with unforeseen elements in what has been deemed an ‘artful process’.^5^

The insightful paper ‘ADAPTing to participant distress: A protocol for applied improvisation in healthcare,’ contained in this issue,^6^ clarifies interventions that can secure a safe learning environment amid distress when using applied improvisation. The article presents us an inspiring perspective, as detailed below, and points us to the boundless potential of improvisation.

Improvisation helps to shape our attitude in dealing with the complexity of the provision of care.

Before getting to that though, it is important to consider how the article paradoxically links improvisation and protocol together. Isn't doing so contrary to the core elements of both? How can improvisation (focused on the process of working with uncertainty and complexity) and protocol (a process of following predictable steps) be united? A closer look into ways to study a butterfly might help clarify how we have come to think about this paradox. Researching the wonders of the butterfly can be done in several ways. One can study its behaviour, such as movement of the wings and flying patterns. One can also study its characteristics through dissection, measuring the weight and the size of individual wings or legs, for example. Where the first approach focuses on the process, the second focuses on more physical parameters. Even though both approaches may be valid for different purposes, it is obvious that dissecting the butterfly makes it such that the process of flying cannot be studied anymore.

The same goes for improvisation. Metaphorically, a protocol for improvisation seems analogous to the hand of the researcher clipping the butterfly's wings and preventing it from actually flying. That said, a protocol, if used as an inspirational guide, may facilitate improvisation by helping the butterfly understand how to fully expand its wings to take flight. Protocols, like ADAPT, may bring confidence to teachers who are unfamiliar with improvisation as a method just as readily as they strive to provide trainees with crucial skills. Used as a platform for exploration, a protocol does not have to be contrary to improvisation, but may enhance enchantment with the collaborative effort required to undertake improvisation.

Focus on improvisation as a process helps us cultivate a perspective of wonder.

With that recognized, the boundless potential of improvisation lies in its ‘enchantment’, drawing pleasure from something beautiful, despite distress being deemed a necessary, maybe even crucial, element. The word emotion derives from the verb ‘emovere’, that ‘which moves out’. Emotions, therefore, have much to do with that which sets us in motion.^7^ Discomfort and distress, as emotions, are elements that add to the dynamic of learning. However, the balance between discomfort as a facilitating force that ‘motivates’ towards improvement and distress that paralyses and hampers learning is a delicate one. Experiencing emotions and their influence on performance is an important part of becoming comfortable with the situations that demand improvisation; letting emotions run wild, however, without reflection and debriefing, may trigger trauma and detachment. Virtue is in the middle, as Aristotle concluded.

It is helpful, therefore, that the ADAPT protocol distinguishes between different types of distress, each accompanied by a different approach (with phases and steps). At the same time, it is valuable to recognize that the concept overarching them all is ‘relationality’. The protocol illustrates how the emotions of all individuals involved can be valued and the learning process of the group can be secured, through the active engagement of a facilitator, by reciprocal processes of attuning to one another. That is key, we argue, to the improvisational learning process, both for the individual and the group alike. Attuning to each other underscores that all are seen and that the contribution of everybody is valued. Relational safety must be built. It is the act of opening up, being seen, seeing the other, and working to coordinate movements together that creates safety rather than that safety can be secured prior to shared experience. In other words, it is the sense of wonder created through relationality in collaboration that spurs us to curiosity about our colleagues and becomes the driving force in shaping safety and enchantment with what can be achieved through improvisation.

Engaging with emotions can build relational safety and enlarge our collaborate sense of enchantment.

In sum, establishing a sense of wonder with the situation, and with collaborators, enables one to reap the benefits of improvisation outside the medical education setting. In a world full of complexity, improvisation will be necessary when facilitators of a formal process are not present. That makes it critical that we both practice a protocol of improvisation within medical education and engage with emotions as a normal part of those learning experiences. It is only through the enchantment of collaborative improvisation that one can make productive and generative improvisation a routine part of their daily practice. Leaving healthcare providers to be confronted with the complexities of the unforeseen only after entering the workplace can be baffling, causing the types of discomfort and distress that are more likely to cause conflict within teams that are stymied by uncertainty about what strategies to pursue.

Improvisation provides a potent attitude that can greatly contribute to daily clinical practice as well.

We want, instead, to encourage the experience of distress during learning that enables us to use ADAPT. We have an obligation, in both education and daily professional practice, to shape of relational safety, which leads us to propose to complement ADAPT with SPACE. For improvisation is shaping of a Secure Place for Attunement and Collaborative Enchantment.
